# A rare case of tension pneumoperitoneum secondary to gastric perforation associated with severe aortic compression

**DOI:** 10.1259/bjrcr.20210090

**Published:** 2021-10-05

**Authors:** Davyd Greenish, Samir Pathak, Daniel Titcomb, Lynne Armstrong

**Affiliations:** 1 University Hospitals Bristol and Weston, Bristol, UK; 2 University Hospitals Bristol and Weston, University of Bristol, Bristol, UK

## Abstract

A 36-year-old male was critically unwell with acute central abdominal pain and distension. CT demonstrated severe pneumoperitoneum leading to compression and total occlusion of the inferior vena cava and occlusion of the aorta. At laparotomy, a perforated posterior gastric ulcer was found with four quadrant contamination. A damage control procedure was performed and a re-look laparotomy was carried out 2 days later where bowel ischaemia was found. Despite being supported on the intensive care unit, unfortunately the patient died. Tension pneumoperitoneum leading to occlusion of the aorta is very rare and the severity of this condition should be recognised; it has never been survived in the reported literature. Rapid assessment and investigation is essential to ensure the timely treatment of this disease.

## Clinical presentation

A 36-year-old male was admitted to the emergency department critically unwell with a distended abdomen and central abdominal pain for the preceding 3 h. His blood pressure on admission was 136/77 mmHg and his lactate was raised at 14 mmol l^−1^ (0.5–2.2 mmol l^−1^). Examination revealed a peritonitic abdomen and loss of femoral pulses bilaterally.

## Differential diagnosis

The differential diagnosis prior to imaging included pneumoperitoneum secondary to gastric or bowel perforation. Vascular compromise including type B aortic dissection or type A aortic dissection with extension was also considered due to the lack of femoral pulses. Imaging with CT was promptly requested and performed due to the diagnostic uncertainty.

## Investigations

**Figure 1.**
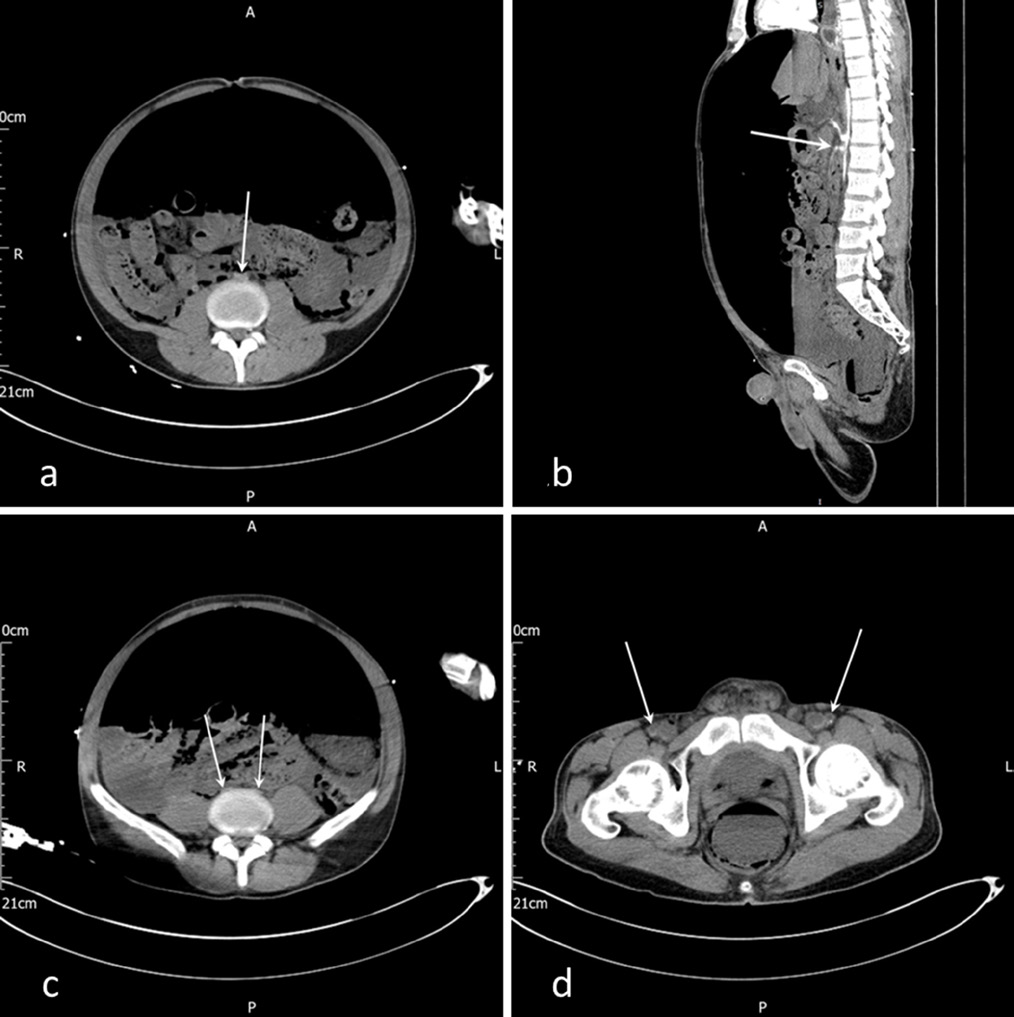
(a) Axial slice of an arterial phase CT scan at the level of the L4 superior endplate with a white arrow showing occlusion of the abdominal aorta (b). Sagittal slice of an arterial phase CT scan demonstrating minimal opacification of the superior mesenteric artery (white arrow) (c). Axial slice of an arterial phase CT, with white arrows demonstrating bilaterally collapsed common iliac arteries (d). Axial slice of an arterial phase CT, with white arrows showing common femoral arteries of normal calibre but with almost no contrast contained within them

Arterial and portal venous phase CT of the abdomen and pelvis extending to the mid-thigh was performed. The scans demonstrated extensive free fluid within the abdomen and severe pneumoperitoneum leading to compression of the inferior vena cava (IVC) and the aorta. There was a short segment aortic occlusion at the level of the L4 superior endplate (Figure 1a). There was minimal opacification with contrast of the superior mesenteric artery (Figure 1b) as well as the iliac and femoral arteries (Figure 1c and d). The portal venous phase CT demonstrated poor opacification of the venous system, due to reduced venous return secondary to high intra-abdominal pressures. Gas in the portal vein and splenic parenchyma raised the suspicion of bowel ischaemia. There was no pneumatosis intestinalis seen.

The normal blood pressure on presentation, acute (3 h) onset of abdominal pain and rapid clinical deterioration coupled with the initial imaging findings of compression of the aorta with a rapid transition point as it descended through the hiatus into the abdomen support the diagnosis of aortic compression secondary to tension pneumoperitoneum.

**Figure 2.**
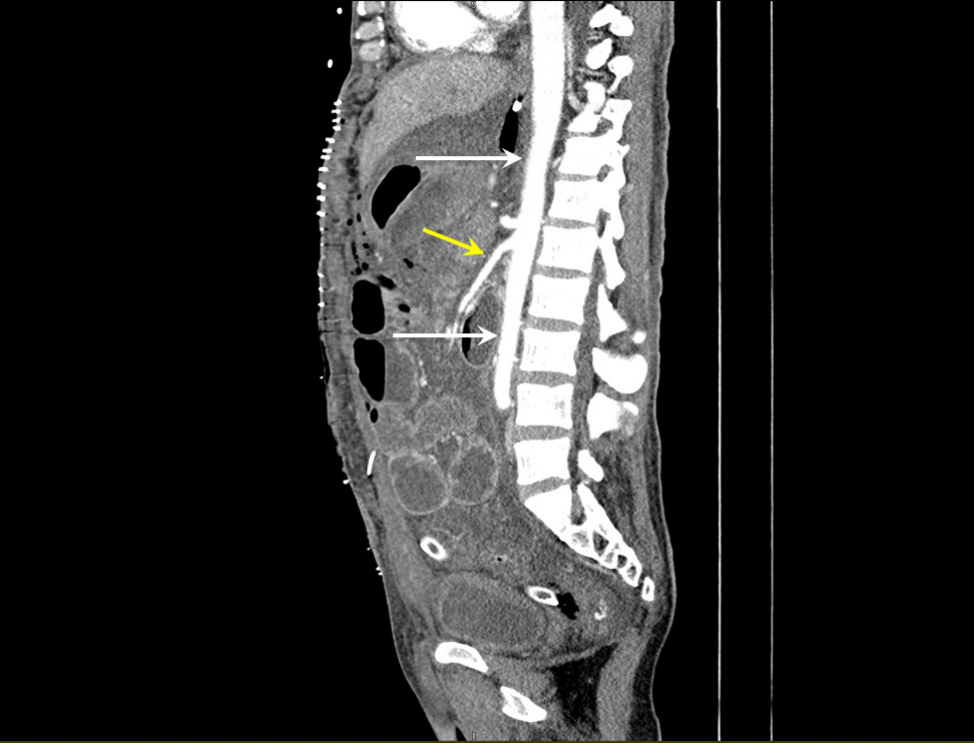
Sagittal slice of an arterial phase CT scan, demonstrating return to normal opacification of the abdominal aorta (white arrows) and the superior mesenteric artery (yellow arrow).

6 days post-treatment, a follow-up CT of the abdomen and pelvis showed normal appearances of the opacified aorta (Figure 2).

## Treatment

The patient was taken to theatre for an emergency laparotomy where a perforated posterior gastric ulcer with four quadrant contamination was found. There was no bowel ischaemia found at initial laparotomy. Femoral pulses returned following surgical decompression of the abdomen. The ulcer was biopsied and closed and the abdomen was washed out; the abdomen was not closed and a plan for a re-look laparotomy was made for the following 24–48 h. When the patient returned to theatre 2 days later for the re-look laparotomy with the intention of closing the abdomen, the right colon was found to be ischaemic. A partial small bowel resection and right hemicolectomy had to be undertaken. The cause for the bowel ischaemia was possibly secondary to a combination of superior mesenteric arterial and venous compromise.

## Outcome and follow-up

Tension pneumoperitoneum (TP) itself is a rare condition; cases reaching pressures that lead to compromise of the arterial supply to the abdominal organs and lower limbs are extremely rare. Initially, the patient performed well following decompression and subsequent surgical interventions to remove ischaemic bowel. He was cared for in the intensive care unit. Unfortunately, the patient died 24 days following his initial admission, secondary to haemorrhagic complications from his abdominal ischaemia.

## Discussion

TP refers to the build-up of gas within the abdominal cavity leading to reduced venous return and haemodynamic instability. It was first described by Oberst in 1917 after a patient developed TP following a gastric perforation.^1^ The pathophysiology of this condition is believed to involve a ‘ball and valve’ mechanism, whereby air can enter the abdominal cavity (commonly via a perforation along the gastro-intestinal tract) but cannot escape via the same route, leading to an increase in intra-abdominal pressures.^2,3^

TP is a rare condition, but has been reported several times in the literature causing reduced venous return.^4^ Pathological intra-abdominal pressures are considered to be >12 mmHg and the pressure required to cause organ failure is >25 mmHg.^4^ In extremely rare cases, intra abdominal pressures may rise far enough (100 mmHg in canine models) to cause aortic compression and occlusion.^5^ Following an extensive literature search, we believe that there have only been four previous reports of TP leading to raised intra abdominal pressures high enough to cause compression of the aorta. In all of these cases, the patients died shortly after presentation.

Treatment of TP is concerned with decompressing the abdomen. This can be achieved with various techniques from cannula decompression to percutaneous catheter insertion and ultimately laparotomy.^6,7^

To our knowledge, TP and aortic compression caused by perforated peptic ulcer has only been previously reported once, and this may be the first case causing TP secondary to a gastric ulcer. The patient described in our report has survived longer than any previously reported patient following aortic occlusion secondary to TP. Early diagnosis and treatment is key in these cases if patients are to survive.

## Learning points

1. TP can lead to pressures that are high enough to cause aortic collapse and compression. When this occurs it is associated with a very high mortality rate.
2. Patients presenting with tension pneumoperitoneum should be given the highest priority for emergency surgery, in order to decompress the abdomen, address any contamination, attempt to treat the cause of the pneumoperitoneum and transfer to the intensive care unit swiftly.
3. There should be a high index of suspicion for the presence of ischaemic bowel if patients with tension pneumoperitoneum are taken to theatre for re-look procedures.
4. Physiological processes can be assessed with the use of CT; this case demonstrated that, despite a portal venous timed scan, venous return was not occurring due to high intra-abdominal pressures.
5. Radiologists should consider vascular compromise early when pneumoperitoneum is seen, in order for rapid communication of serious findings such as aortic compression to be made to the surgical team and early treatment to be possible.

## References

[1] Oberst A. Das spannungspneumoperitoneum. Zentralbl Chir 1917; 44: 354–5.

[2] Chan SY, Kirsch CM, Jensen WA, Sherck J. Tension pneumoperitoneum. West J Med 1996; 165(1-2): 61–4. 8855695 PMC1307551

[3] Cadena M, Solano J, Caycedo N, Gomez D, Vinck EE, Quiroga P, et al. Tension pneumoperitoneum: case report of a rare form of acute abdominal compartment syndrome. Int J Surg Case Rep 2019; 55: 112–6. doi: 10.1016/j.ijscr.2019.01.014 30716704 PMC6360270

[4] Kirkpatrick AW, Roberts DJ, De Waele J, Jaeschke R, Malbrain MLNG, De Keulenaer B, et al. Intra-abdominal hypertension and the abdominal compartment syndrome: updated consensus definitions and clinical practice guidelines from the world society of the abdominal compartment syndrome. Intensive Care Med 2013; 39: 1190–206. doi: 10.1007/s00134-013-2906-z 23673399 PMC3680657

[5] Olinde AJ, Carpenter D, Maher JM. Tension pneumoperitoneum. A cause of acute aortic occlusion. Arch Surg 1983; 118: 1347–50. doi: 10.1001/archsurg.1983.01390110089020 6639345

[6] Paschold M, Gockel I, Oberholzer K, Lang H, Düber C. Pneumoabdomen with abdominal compartment and aortic collapse due to gastric bursting acute release by trocar insertion. Circulation 2013; 127: 417–8. doi: 10.1161/CIRCULATIONAHA.112.131763 23339096

[7] Roberts DJ, Ball CG, Kirkpatrick AW. Increased pressure within the abdominal compartment: intra-abdominal hypertension and the abdominal compartment syndrome. Curr Opin Crit Care 2016; 22: 174–85. doi: 10.1097/MCC.0000000000000289 26844989

